# Tripeptides From Casein Are Signal Molecules to Induce the Expression of the Extracellular Protease MCP-01 Gene in Marine Bacterium *Pseudoalteromonas* sp. SM9913

**DOI:** 10.3389/fmicb.2019.01354

**Published:** 2019-06-26

**Authors:** Xiu-Lan Chen, Jin-Yu Yang, Xiao-Yu Zheng, Qi Sheng, Lei Wang, Yu-Zhong Zhang, Qi-Long Qin, Xi-Ying Zhang

**Affiliations:** ^1^State Key Laboratory of Microbial Technology, Marine Biotechnology Research Center, Shandong University, Qingdao, China; ^2^Institute of Agro-Food Science and Technology, Shandong Academy of Agricultural Sciences/Key Laboratory of Agro-Products Processing Technology of Shandong Province/Key Laboratory of Novel Food Resources Processing, Ministry of Agriculture and Rural Affairs, Jinan, China; ^3^Laboratory for Marine Biology and Biotechnology, Qingdao National Laboratory for Marine Science and Technology, Qingdao, China; ^4^College of Marine Life Sciences, Ocean University of China, Qingdao, China

**Keywords:** signal molecule, extracellular protease, induce, marine bacteria, tripeptide

## Abstract

Microbial extracellular proteases play crucial roles in marine protein degradation and nitrogen recycling. Although a large number of marine bacteria are found to produce extracellular proteases, it is still unknown how marine bacteria respond to environmental proteins to activate the expression of genes encoding extracellular proteases. The inducing signal molecule for marine bacterial extracellular proteases has never been identified. In this study, we identified tripeptides as the inducing signal molecules for the extracellular protease MCP-01 of the deep-sea bacterium *Pseudoalteromonas* sp. SM9913. We found that casein, but not casamino acids, can induce the gene expression and synthesis of MCP-01, suggesting that peptides rather than amino acids derived from casein induce the gene expression and synthesis of MCP-01 in SM9913. Then, casein was hydrolyzed by SM9913 extracellular proteases, and the peptides with inducing effect were isolated and characterized. Finally, four tripeptides, SPP, RYP, RQF and FRQ, were shown to have significant inducing effect on the expression of MCP-01 gene, indicating that they are likely the inducing signal molecules for the expression of protease MCP-01 gene in SM9913. This study sheds light on the induction mechanism for the gene expression and biosynthesis of marine microbial extracellular proteases, which is helpful in better understanding the adaptation of bacteria to deep-sea sedimental environment.

## Introduction

Proteins are the most abundant organic nitrogen source in the sea, and constitute large fractions of marine organic matters (Thamdrup and Dalsgaard, 2008; Lloyd et al., 2013; Moore et al., 2014). Therefore, the degradation of marine proteins is an important step of marine matter cycling. Extracellular protease-producing microbes are recognized as key players in the microbial degradation of marine organic nitrogen (Herbert, 1999; Xiong et al., 2007; Zhao et al., 2008; Chen et al., 2009; Zhou et al., 2009), which play crucial roles in marine nitrogen recycling. A large number of various marine bacteria that produce extracellular proteases have been reported, which are from different marine environments (Olivera et al., 2007; Xiong et al., 2007; Zhou et al., 2009, 2013; Zhang et al., 2015). For example, Olivera et al. (2007) screened 14 protease-producing strains from sub-Antarctic sediments. Zhou et al. (2009) screened 78 protease-producing bacterial strains from the sediments of the South China Sea. Zhang et al. (2015) screened 66 protease-producing bacteria from the sediments of the eutrophied Jiaozhou Bay, China. Moreover, some extracellular proteases from marine bacteria have been characterized. The extracellular proteases from marine bacteria so far reported are mainly serine proteases and metalloproteases (Zhou et al., 2009, 2013; Zhang et al., 2015). For example, several novel serine proteases of the S8 family have been identified from deep-sea sedimental bacteria and characterized (Zeng et al., 2003; Chen et al., 2007; Yan et al., 2009; Ran et al., 2014), and metalloproteases of the M4 family, the M12 family, and the M23 family also have been identified from deep-sea sedimental bacteria and characterized (Chen et al., 2009; Gao et al., 2010; He et al., 2012; Zhao et al., 2012). However, although a large number of protease-producing bacteria and extracellular proteases have been studied, it is still unknown how marine bacteria respond to environmental proteins to initiate the gene expression, biosynthesis and secretion of extracellular proteases.

Though the presence of outside proteins can induce the expression and secretion of microbial extracellular proteases, it is generally thought that proteins are not a direct inducing signal molecule. Instead, small molecules derived from proteins, such as amino acids and oligopeptides, are likely inducing signal molecules. However, to date, the signal molecules to induce the gene expression and biosynthesis of extracellular proteases of marine bacteria have never been reported.

*Pseudoalteromonas* sp. SM9913 (SM9913 hereafter) is a protease-producing bacterium isolated from a deep-sea sediment, and protease deseasin MCP-01 is the most abundant protease secreted by this strain (Chen et al., 2003). Deseasin MCP-01 has been well characterized to be a serine protease of the S8 family and has caseinolytic and collagenolytic activity (Chen et al., 2007; Zhao et al., 2008; Ran et al., 2013). Moreover, the genomic DNA of strain SM9913 has been sequenced (GenBank accession No. GCA_000184065.1), and the strategies employed by this strain to adapt to the cold deep-sea sedimental environment have also been studied (Qin et al., 2011; Mi et al., 2015). These studies lay a good foundation for studying the signal molecules that induce the gene expression and biosynthesis of the extracellular protease MCP-01 in strain SM9913 in response to outside proteins.

In this study, we aimed to identify the signal molecules that induce the expression of the gene (*mcp01*) encoding the extracellular protease MCP-01 in SM9913. We found that casein in the medium could induce the gene expression and synthesis of MCP-01, but the casamino acids could not, indicating that peptides rather than amino acids derived from casein could serve as the molecules that trigger the expression of gene *mcp01* in SM9913. Then, we isolated and identified the peptides with inducing effect on the expression of gene *mcp01* from the hydrolysate of casein that was hydrolyzed by the extracellular proteases of SM9913. The results showed that four tripeptides had significant inducing effect on the expression of gene *mcp01*, indicating that they are potential inducing molecules for the expression of gene *mcp01* in SM9913.

## Materials and Methods

### Materials, Strains, and Media

SM9913 was originally isolated from a deep-sea sediment (Chen et al., 2003). Bovine casein was purchased from Sigma-Aldrich chemical Co. (US), and casamino acids from Sangon Biotech (Shanghai) Co., Ltd. (China). Dipeptides and tripeptides were synthesized by Shanghai Jiepi Biotechnology Co., Ltd. (China). The casein medium (pH 7.5) contained 0.5% (w/v) casein, 1% (w/v) mannitol and artificial seawater. The casamino acids medium (pH 7.5) contained 0.5% (w/v) casamino acids, 1% (w/v) mannitol and artificial seawater.

### Analysis of the Extracellular Protease Activity of SM9913 Cultured With Casein or Casamino Acids

SM9913 was cultivated at 15°C, 180 rpm in the casein medium and the casamino acids medium. After being cultivated for 24, 36, 48, 56, and 60 h, the cultures were sampled and the samples were centrifuged at 10,000*g*, 4°C for 20 min. The supernatants were collected for extracellular protease activity analysis. Protease activity was measured by the Folin-phenol method as previously described (Chen et al., 2003).

### Secretome Analysis of the Production of Extracellular Proteases of SM9913 Cultured With Casein

SM9913 was cultured at 15°C, 180 rpm in the casein medium for 24 h. After cultivation, the fermentation broth was centrifuged at 4°C, 10,000*g* for 10 min. The proteins in the supernatant were extracted as previously described (Wang et al., 2019). The extracted proteins were digested by trypsin (Promega, US) with an enzyme-to-substrate ratio of 1:100 (w/w) at 37°C for 12 h. The digested proteins were desalted with C_18_ Ziptip according to the manufacturer’s instruction (Millipore, Germany), and then subjected to nanoscale LC-MS/MS analysis. The data were analyzed with Mascot software (Thermo Fisher Scientific, US).

### Hydrolysis of Casein by the Extracellular Protease of SM9913

After SM9913 was cultivated at 15°C, 180 rpm in the casein medium for 3 days, the culture was centrifuged at 10,000*g* and 4°C for 20 min. The supernatant was collected for casein hydrolysis. A mixture of 2% (w/v) casein and the supernatant (1:1, v/v) was prepared, and incubated at 40°C and 100 rpm. After 2 h of incubation, the hydrolytic reaction was stopped by incubating the mixture at 95°C for 10 min. Then, the mixture was centrifuged at 12,000*g* and 4°C for 30 min, and the supernatant was collected as casein hydrolysate.

### Separation of Peptides in the Casein Hydrolysate by Gel Filtration

To remove the peptides larger than 2 kDa, the casein hydrolysate was ultrafiltrated by a membrane with a molecular cut off of 2 kDa. The permeate solution was concentrated by lyophilization and loaded on a Sephadex G15 column (1.6 cm × 100 cm) that was eluted with distilled water at a speed of 0.30 ml/min and monitored at 220 nm. Fractions of each peak were collected and lyophilized.

### Identification of Peptide Sequences

The molecular masses of the peptides from peaks 4, 5, and 6 were determined by MALDI-TOF-MS. The sequences of the peptides were determined by MASCOT MS/MS Ion Research and Expasy tools^1^.

### Analysis of the Transcription Level of *mcp01* by Real-Time qPCR

The resting cells of SM9913 were used in the induction of *mcp01* expression, which were prepared as follows. SM9913 was cultured in the casamino acids medium at 20°C and 180 rpm. When the OD_600_ of the culture reached 0.4 ~ 0.5, the cells were collected by centrifugation, and washed three times with sterilized seawater, which were then shaken at 15°C and 180 rpm for 20 h. After being shaken, the cells were washed twice with sterilized seawater, and then incubated at 4°C for 24 h. After incubation, the cells were collected by centrifugation and resuspended in sterilized seawater, which were used as the resting cells. To induce the expression of *mcp01*, the resting cells of SM9913 were inoculated into the casamino acids medium, the casein medium, the medium containing 1% mannitol and 0.5% casein peptides from each peak and the medium containing 1% mannitol and 2 mM of each tripeptide, respectively, and incubated at 15°C and 180 rpm for a certain time. Total RNA was extracted with RNeasy Protect Bacteria Mini Kit (QIAGEN, Germany) according to the manufacturer’s protocol, and further treated with PrimeScript™ RT reagent Kit with gDNA Eraser (Perfect Real Time) (Takara, Japan) to remove the genomic DNA. Reverse transcription was conducted according to the manufacturer’s instructions. The obtained cDNA was used as template for qPCR. The qPCR was conducted in triplicate using the SYBR^®^ *Premix Ex Taq*™ (Tli RNaseH Plus) (TaKaRa, Japan) on a LightCycler^®^480 (Roche, Switzland), with 10 μM of the forward and reverse primers and 100 ng of cDNA. Data analysis was carried out using the LightCycler^®^480 software with “Advanced Relative Quantification” method and was normalized to the endogenous control *rpoD* with expression at 0 h as the reference sample. The primers used in this study are shown in Table 1.

**Table 1:** Primers for the reference gene and the target gene in Real-time qPCR.

Primer name	Primer sequence (5′–3′)
*rpoD*-F	CGCATATTATTGACTGGTTAGGTG
*rpoD*-R	CAAGGGTTGAGGGTTCATAGC
*mcp01*-F	AGCGACTGTAGGTGATACGGTTAC
*mcp01*-R	CGGACGAGCGGTAGTTGCG

## Results

### Gene Expression and Synthesis of MCP-01 Is Induced by Casein But Not Casamino Acids

**Figure 1:**
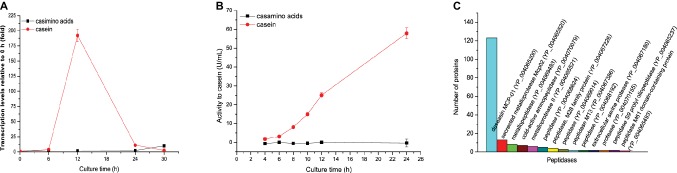
Gene expression and biosynthesis of protease MCP-01 in SM9913 cultured in the medium containing casamino acids or casein as the sole nitrogen source. **(A)** Expression of gene *mcp01* analyzed by RT-qPCR. Relative gene expression levels were normalized to 1 before induction (0 h). Expression levels of *rpoD* were used as an endogenous control in all samples. Values are the mean of three biological replicates. *Error bars* are the S.D. from these replicates. **(B)** The extracellular protease activity of SM9913. The protease activity was measured with casein as substrate. **(C)** Secretome analysis of the production of extracellular proteases of SM9913 cultured for 24 h in the medium containing casein as the sole nitrogen source.

To investigate the inducing molecules for the expression of gene *mcp01* in SM9913, we compared the inducing effects of casein and casamino acids on the transcription level of gene *mcp01* in SM9913 by RT-qPCR. In order to eliminate the disturbance of intracellular amino acids, we used resting cells in this experiment because intracellular amino acids have been used up in resting cells. The resting cells of SM9913 were incubated at 15°C in a medium containing 0.5% casein or 0.5% casamino acids as nitrogen source and mannitol as carbon source, and the transcription levels of gene *mcp01* in the cells were detected by RT-qPCR after incubation for 6, 12, 24 and 30 h. As shown in Figure 1A, when casein was present in the medium as the sole nitrogen source, the transcription of gene *mcp01* in SM9913 was induced, and its level increased by approximately 200 folds at the 12th h, and then fell to 11 folds at the 24th h. This indicates that casein in the medium as the sole nitrogen source has inducing effect on the transcription of gene *mcp01.* In contrast, when casamino acids was present in the medium as the sole nitrogen source, the transcription level of gene *mcp01* in SM9913 resting cells did not increase significantly, indicating that casamino acids has little inducing effect on the transcription of *mcp01*. Correspondingly, extracellular protease activity was detected in the medium containing casein, but not in the medium containing casamino acids (Figure 1B), and our secretome analysis showed that protease MCP-01 in the culture of SM9913 is predominant over other secreted proteases (Figure 1C), indicating that the extracellular protease activity in the culture of strain SM9913 mainly resulted from MCP-01. Taken together, these results indicate that casein can induce the gene expression, and therefore synthesis and secretion of extracellular protease MCP-01, but amino acids derived from casein cannot.

### Identification of the Fragments with Inducing Effect on MCP-01 Synthesis From the Hydrolytic Products of Casein by Gel Filtration

**Figure 2:**
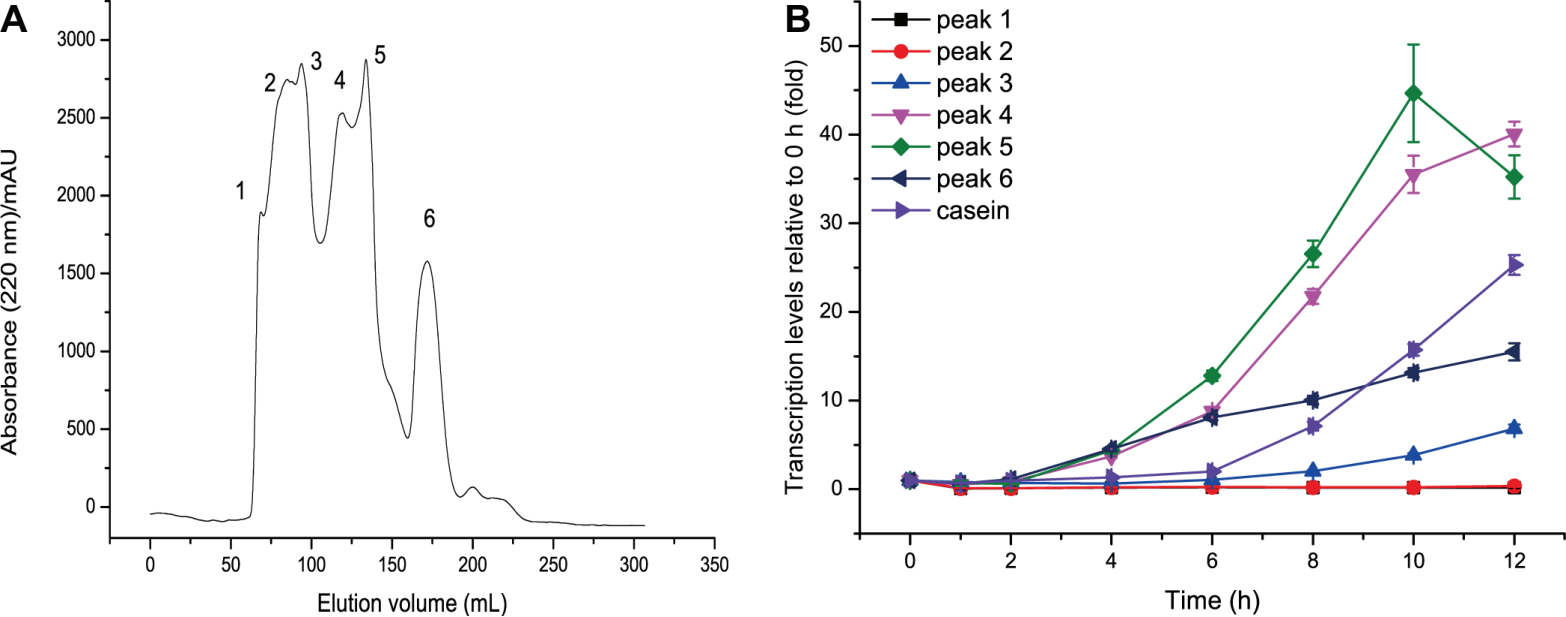
Gel filtration of casein hydrolysate **(A)** and effects of the fractions from casein hydrolysate on the expression of *mcp01* **(B)**. Gene expression of *mcp01* was conducted by RT-qPCR. Values are the mean of three biological replicates. *Error bars* are the S.D. from these replicates.

It is generally considered that proteins such as casein are not the molecules that directly induce extracellular protease synthesis. Instead, some small molecules derived from proteins are the true inducing molecules. Small molecules derived from casein hydrolysis include amino acids and peptides. Since amino acids derived from casein had no inducing effect on MCP-01 synthesis, it is likely that peptides derived from casein hydrolysis are the true inducing molecules for MCP-01 synthesis. Therefore, we hydrolyzed casein with the extracellular proteases from SM9913 cultured in the medium containing casein as the sole nitrogen source. Peptides with a molecular mass higher than 2 kDa were removed from the hydrolysate by ultrafiltration, and the remaining with a molecular mass less than 2 kDa were further fractioned by gel filtration chromatography on a Sephadex G15 column. After gel filtration, six peptide fractions were obtained (Figure 2A). Then, the inducing effects of the peptides in the six fractions on the transcription of *mcp01* in SM9913 were analyzed. As shown in Figure 2B, the peptides in peaks 4, 5, and 6 have significant inducing effect on the transcription of *mcp01*, whereas peptides in peaks 1, 2, and 3 had little effect. This suggests that small peptides have better inducing effect than big peptides.

### Identification of the Dipeptides and Tripeptides Common in Peaks 4, 5, and 6

**Figure 3:**
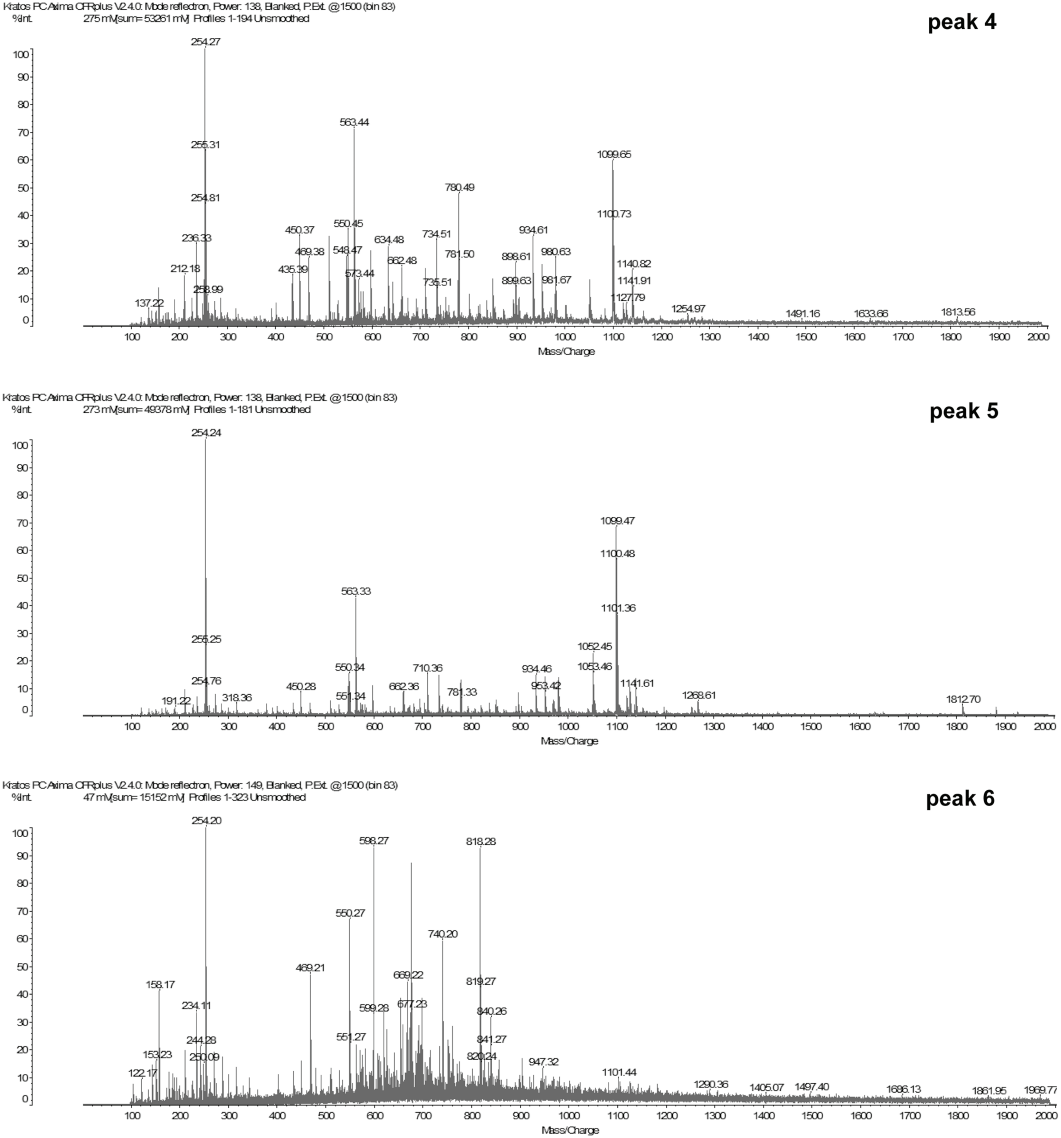
MALDI-TOF analysis of the molecular weights of peptides in peaks 4, 5, and 6.

The result in Figure 2B suggested that some small peptides in peaks 4, 5, and 6 have inducing effect on *mcp-01* transcription. To identify the sequences of these inducing peptides, we first determined the molecular masses of the peptides in peaks 4, 5 and 6 by MS (MALDI-TOF). As shown in Figure 3, the molecular masses of the peptides in peaks 4 are in the range of 200–1,100 Da, those in peak 5 are in the range of 250–1,100 Da, and those in peak 6 are in the range of 150–300 Da and 400–850 Da. Considering that small peptides are likely the inducing molecules, we identified the sequences of dipeptides and tripeptides with a molecular mass less than 650 Da in peaks 4, 5, and 6 by ExPasy according to the sequences of bovine *α*-casein (S1, S2), *β-*casein and *κ*-casein. As a result, 190 dipeptide and 68 tripeptide sequences were identified in peak 4 (Supplementary Table S1), 74 dipeptide and 63 tripeptide sequences in peak 5 (Supplementary Table S2), and 52 dipeptide and 34 tripeptide sequences in peak 6 (Supplementary Table S3). Considering that the peptides co-existing in these three peaks are most likely the inducing molecules for MCP-01 synthesis, we further identified the dipeptide and tripeptide sequences common in peaks 4, 5, and 6. There were only one dipeptide sequence (HV) but eight tripeptide sequences common in peaks 4, 5, and 6 (Table 2).

**Table 2:** Tripeptides common in peaks 4, 5, and 6.

No.	Sequence (N → C)	Location
(1)	(F)ALP(Q)	*α*-S2-casein
(2)	(F)RQF(Y)	*α*-S1-casein
(3)	(L)FRQ(F)	*α*-S1-casein
(4)	(L)RFF(V)	*α*-S1-casein
(5)	(E)SPP(E)	*κ*-casein
(6)	(M)AIP(P)	*κ*-casein
(7)	(S)RYP(S)	*κ*-casein
(8)	(E)RFF(S)	*κ*-casein

### Tripeptides Have Inducing Effect on *mcp-01* Transcription

**Figure 4:**
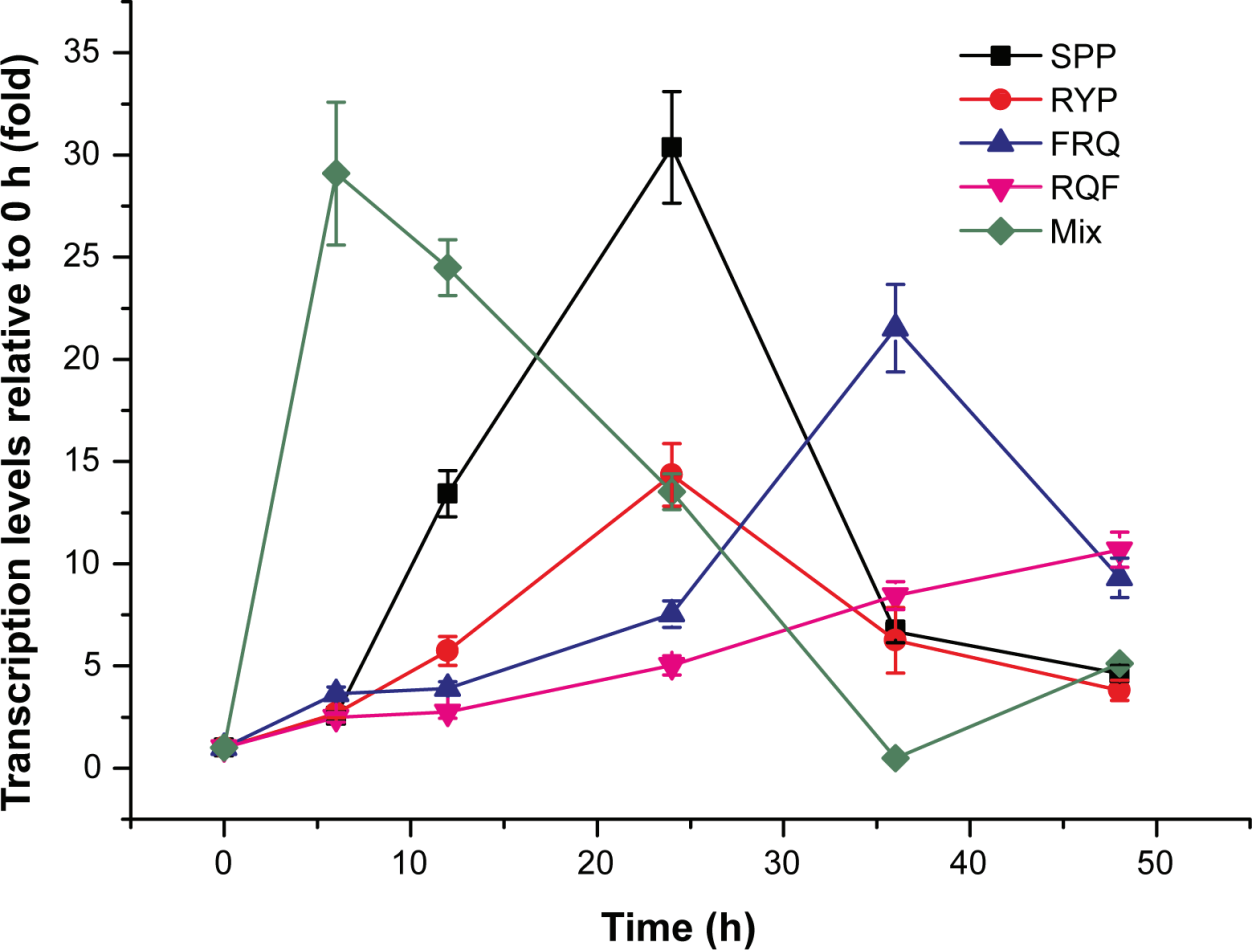
Expression of gene *mcp01* induced by the tripeptides identified from casein hydrolysate. Mix, the mixture of the four tripeptides SPP, RYP, FRQ, and RQF (2 mM each). The concentration of each tripeptide used for induction was 2 mM. Gene expression of *mcp01* was conducted by RT-qPCR. Values are the mean of three biological replicates. *Error bars* are the S.D. from these replicates.

To find which peptides are the true inducing signal molecules for MCP-01 synthesis, the dipeptides and tripeptides common in peaks 4, 5, and 6 were synthesized and their inducing effects on *mcp-01* transcription in SM9913 were detected. The result showed that four tripeptides, SPP, RYP, RQF, and FRQ, had significant inducing effect on *mcp01* transcription in SM9913. The inducing effects of these tripeptides are different. Among them, SPP had the strongest inducing effect, and RQF had the least. The order of the inducing effects of the four tripeptides is SPP > RYP > FRQ > RQF (Figure 4). This result indicated that these tripeptides are likely inducing signal molecules to initiate MCP-01 synthesis in SM9913, especially SPP and RYP.

To investigate whether the four tripeptides combined have a stronger inducing effect on *mcp01* transcription than individual tripeptide, we analyzed the inducing effects of the four tripeptides altogether on *mcp01* transcription in SM9913. The result showed that, when a mixture of the four tripeptides was used, peak level of *mcp01* transcription was detected at the 6th h, a time earlier than those induced by individual tripeptide, with a level comparable to that induced by SPP, the most effective tripeptide (Figure 4). Thus, a cumulative effect was not observed.

## Discussion

Both amino acids and oligopeptides have been reported to be capable of inducing the biosynthesis of extracellular proteases of terrestrial microbes. Homma et al. (1993) reported that the presence of only casamino acids in the medium induced the production of extracellular aspartyl proteases in *C. albicans*. Martínez and Ljungdahl (2005) also reported that the presence of low concentrations of extracellular amino acids provides the initiating signals for the functional expression of secreted aspartyl proteases in *C. albicans*. In addition, the synthesis of *Bacillus* extracellular proteases was reported to be induced by amino acids at a substantially decreased concentration in the cells at the exponential growth phase (Egorov et al., 1983; Barbieri et al., 2016). On the other side, the peptidase genes *prtP* and *prtM* of *Lactococcus lactis* SK11 were reported to be controlled at the transcriptional level by specific dipeptides (Marugg et al., 1995). Lerner and Goldman (1993) reported that peptides containing eight or more residues in length induced protease production in *Candida albicans*. Therefore, it seems that both amino acids and oligopeptides are likely the inducing signal molecules for the synthesis of microbial extracellular proteases. However, the exact amino acids or peptides are still rarely identified.

Most deep-sea sediments are underexplored habitats, and our knowledge on how bacteria adapted to deep-sea sediments is still limited. Deep-sea bacteria that secrete extracellular proteases are a key player to degrade organic nitrogen and drive nitrogen cycling in deep sea (Herbert, 1999; Xiong et al., 2007; Zhao et al., 2008; Chen et al., 2009; Zhou et al., 2009). Therefore, it is important to study how deep-sea bacteria regulate the gene expression and biosynthesis of their extracellular proteases in response to outside protein source. Identification of the environmental signal molecules to initiate the gene expression and biosynthesis of extracellular proteases is essential for clarifying how deep-sea bacteria regulate the gene expression and biosynthesis of their extracellular proteases. However, although a large number of marine sedimental bacteria have been found to produce extracellular proteases (Xiong et al., 2007; Zhou et al., 2009, 2013; Zhang et al., 2015), it is still unknown what kinds of amino acids or oligopeptides are signal molecules to induce the synthesis of microbial extracellular proteases in the sea.

In this study, we found that casein can induce the gene expression and synthesis of the extracellular protease MCP-01 of the deep-sea bacterium SM9913, but the casamino acids cannot. This indicates that oligopeptides derived from casein are more likely the signal molecules to induce the synthesis of MCP-01 in SM9913 than amino acids. Therefore, to find the casein-derived oligopeptides that have inducing effect on the expression of MCP-01 gene, we hydrolyzed casein with the extracellular proteases from SM9913, and identified the oligopeptides that have the inducing effect from casein hydrolysate. Finally, we found that several tripeptides, including SPP, RYP, RQF and FRQ, can significantly induce the production of protease MCP-01 in SM9913, which indicates that these tripeptides are most likely the inducing signal molecules for the synthesis of MCP-01 in SM9913. In addition, we noticed that the four tripeptides did not show a cumulative effect when they were used together and that their inducing effect was far less than that of casein. It is possible that some peptides that have inducing effect on *mcp01* transcription were unable to be identified from the casein hydrolysate because we just tested the tripeptides common in peaks 4, 5, and 6 (Figure 2) and did not test the others due to the large number of the others (Supplementary Tables S1–S3).

Lerner and Goldman (1993) have investigated the characters of the peptides to induce the production of the extracellular proteases of *C. albicans*, and drew several conclusions. They found that: (1) peptides of eight or more residues in length induced protease production in *C. albicans* while peptides of seven or fewer residues did not; (2) all peptides that induced protease production contain the dipeptide sequence (His/Lys)-Pro and lack Gly residues, however, the dipeptide sequence (His/Lys)-Pro is not necessary; (3) all non-inducing peptides, larger than dipeptides, contain at least one Gly residue and lack the dipeptide (His/Lys)-Pro motif, suggesting that the sensory mechanism in *C. albicans* may detect the side chains at each residue in the inducing peptide. Consistent with the findings of Lerner and Goldman (1993), none of the four tripeptides (SPP, RYP, RQF, and FRQ) that have inducing effect on the biosynthesis of MCP-01 in SM9913 contain the Gly residue, suggesting that the sensor in SM9913 may also detect the side chains at each residue in the inducing peptide, which needs further study.

To find information on the transcriptional regulation of gene *mcp01* in SM9913, we analyzed the gene organization in the surrounding of gene *mcp01*. As shown in Supplementary Figure S1. The genes upstream *mcp01* include two genes encoding hypothetical proteins, and several genes encoding proteins related to curlin production and assembly. Downstream of *mcp01* are three genes encoding chitinases. Thus, based on annotation of these genes, it appears that the genes surrounding *mcp01* have little relation with the transcription regulation of gene *mcp01.* Therefore, further study on the regulation of the gene expression of MCP-01, such as identification of the sensor in response to the inducing signal molecules, and the signal transmission pathway in SM9913 to initiate *mcp01* transcription is necessary in the future.

In conclusion, in this study, short peptides rather than amino acids were found to induce the gene expression of extracellular protease MCP-01 in the deep-sea bacterium SM9913. Four tripeptides derived from casein hydrolysis, in which all residues have side chains, were shown to have significant inducing effect, which therefore, are potentially the signal molecules to induce the production of protease MCP-01 in SM9913. This study sheds light on the induction mechanism for the synthesis of microbial extracellular proteases in the sea, and is helpful in better understanding the adaptation of bacteria to deep-sea sedimental environment.

## Author Contributions

J-YY, X-YZ, LW, and QS performed all the experiments. X-LC and J-YY wrote the paper. X-LC directed the study. Y-ZZ, Q-LQ, and X-YZ designed the study.

### Conflict of Interest Statement

The authors declare that the research was conducted in the absence of any commercial or financial relationships that could be construed as a potential conflict of interest.

## Footnotes

^1^www.expasy.org
