# Supplementary material for: Tripeptides From Casein Are Signal Molecules to Induce the Expression of the Extracellular Protease MCP-01 Gene in Marine Bacterium Pseudoalteromonas sp. SM9913
Source: Front Microbiol. 2019 Jun 26;10:1354. doi: 10.3389/fmicb.2019.01354 (PMC6606773; doi:10.3389/fmicb.2019.01354)
Supplement: Supplementary file 1 [file Data_Sheet_1.docx]

**Tripeptides from casein are signal molecules to induce the expression of the extracellular protease MCP-01 gene in marine bacterium *Pseudoalteromonas* sp. SM9913**

Xiu-Lan Chen^1,#^, Jin-Yu Yang^1,4,#^, Xiao-Yu Zheng^1^, Qi-Sheng^1^, Lei Wang^1^, Yu-Zhong Zhang^1,2,3^, Qi-Long Qin^1,*^, Xi-Ying Zhang^1,*^

^1^State Key Laboratory of Microbial Technology, Marine Biotechnology Research Center, Shandong University, Qingdao, 266237, China.

^2^Laboratory for Marine Biology and Biotechnology, Qingdao National Laboratory for Marine Science and Technology, Qingdao 266237, China.

^3^College of Marine Life Sciences, Ocean University of China, Qingdao 266003, China.

^4^Institute of Agro-Food Science and Technology, Shandong Academy of Agricultural Sciences/Key Laboratory of Agro-Products Processing Technology of Shandong Province/Key Laboratory of Novel Food Resources Processing, Ministry of Agriculture and Rural Affairs, Jinan 250100, China.

^#^ Jin-Yu Yang and Xiu-Lan Chen contributed equally to this study.

**Supplementary data**

**TABLE S1** Dipeptides and tripeptides identified from peak 4 from the casein hydrolysate

| **Location** | **Dipeptide** | **Dipeptide** | **Dipeptide** | **Dipeptide** | **Dipeptide** | **Tripeptide** | **Tripeptide** |
| --- | --- | --- | --- | --- | --- | --- | --- |
| ***α*-S1 casein** | (K)HP(I) | (K)EK(V) | (L)EQ(L) | (P)MI(G) | (V)PL(G) | (Q)GLP(Q) | (K)EGI(H) |
|  | (H)PI(K) | (V)NE(L) | (E)QL(L) | (I)GV(N) | (L)GT(Q) | (L)LRF(F) | (E)LFR(Q) |
|  | (P)IK(H) | (N)EL(S) | (R)LK(K) | (V)NQ(E) | (P)SF(S) | (L)RFF(V) | (L)FRQ(F) |
|  | (G)LP(Q) | (S)KD(I) | (P)QL(E) | (N)QE(L) | (S)FS(D) | (F)VAP(F) | (F)RQF(Y) |
|  | (P)QE(V) | (E)DQ(A) | (Q)LE(I) | (Q)EL(A) | (D)IP(N) | (P)FPE(V) | (Q)LDA(Y) |
|  | (L)NE(N) | (D)IK(Q) | (L)EI(V) | (L)AY(F) | (N)PI(G) | (E)VFG(K) | (Y)PSG(A) |
|  | (N)EN(L) | (E)EI(V) | (N)SA(E) | (P)EL(F) | (S)EN(S) | (D)IGS(E) | (V)PLG(T) |
|  | (L)RF(F) | (V)EQ(K) | (H)SM(K) | (L)FR(Q) | (S)EK(T) | (G)SES(T) | (D)APS(F) |
|  | (A)PF(P) | (H)IQ(K) | (M)KE(G) | (Y)QL(D) | (M)PL(W) | (E)RYL(G) | (N)PIG(S) |
|  | (P)FP(E) | (Q)KE(D) | (I)HA(Q) | (D)AY(P) | (P)LW | (Q)LLR(L) | (P)IGS(E) |
|  | (G)KE(K) | (Y)LE(Q) | (Q)KE(P) | (G)AW(Y) |  | (L)LRL(K) |  |
| ***α*-S2 casein** | (K)QE(K) | (F)CK(E) | (L)NE(I) | (R)EQ(L) | (K)KI(S) | (E)KNM(A) | (A)EVA(T) |
|  | (Q)EK(N) | (C)KE(V) | (N)EI(N) | (E)QL(S) | (K)FA(L) | (N)LCS(T) | (Q)GPI(V) |
|  | (E)KN(M) | (V)VR(N) | (I)NQ(F) | (E)EN(S) | (A)LP(Q) | (R)NAN(E) | (N)AVP(I) |
|  | (K)NM(A) | (A)NE(E) | (K)FP(Q) | (V)FT(K) | (Y)LK(T) | (Y)SIG(S) | (F)ALP(Q) |
|  | (S)KE(N) | (E)SA(E) | (Y)LQ(Y) | (T)KL(T) | (P)WI(Q) | (S)IGS(S) | (V)IPY(V) |
|  | (K)EN(L) | (V)AT(E) | (G)PI(V) | (E)EK(N) | (W)IQ(P) | (I)GSS(S) | (V)RYL |
|  | (L)CS(T) | (V)KI(T) | (W)DQ(V) | (E)KN(R) | (V)IP(Y) | (S)SSE(E) |  |
|  | (S)TF(C) | (D)DK(H) | (V)PI(T) | (F)LK(K) | (Y)VR(Y) | (S)AEV(A) |  |
| ***β*-casein** | (R)EL(E) | (L)QD(K) | (P)PL(T) | (L)PL(P) | (Y)QE(P) | (P)GEI(V) |  |
|  | (E)LE(E) | (Q)DK(I) | (P)PF(L) | (P)LP(L) | (P)VR(G) | (S)SSE(E) |  |
|  | (E)EL(N) | (D)KI(H) | (F)LQ(P) | (L)PL(L) | (G)PF(P) | (T)EDE(L) |  |
|  | (G)EI(V) | (I)HP(F) | (M)GV(S) | (L)LQ(S) | (P)FP(I) | (Q)SLV(Y) |  |
|  | (I)TR(I) | (H)PF(A) | (V)KE(A) | (Q)PH(Q) | (F)PI(I) | (P)GPI(H) |  |
|  | (I)NK(K) | (P)FA(Q) | (H)KE(M) | (Q)PL(P) |  | (M)GVS(K) |  |
|  | (K)KI(E) | (Y)PF(P) | (M)PF(P) | (P)LP(P) |  | (A)MAP(K) |  |
|  | (K)IE(K) | (P)FP(G) | (P)FP(K) | (M)FP(P) |  | (V)EPF(T) |  |
|  | (I)EK(F) | (G)PI(H) | (E)PF(T) | (V)LP(V) |  | (Q)SVL(S) |  |
|  | (E)EQ(Q) | (S)LP(Q) | (P)FT(E) | (M)PI(Q) |  | (K)AVP(Y) |  |
|  | (D)EL(Q) | (P)QN(I) | (V)EN(L) | (P)IQ(A) |  | (A)FLL(Y) |  |
|  | (E)LQ(D) | (N)IP(P) | (H)LP(L) | (Q)AF(L) |  | (V)LGP(V) |  |
| ***κ*-casein** | QE(Q) | (S)DK(I) | (A)QI(L) | (N)QD(K) |  | (D)ERF(F) | (T)VPA(K) |
|  | (Q)EQ(N) | (D)KI(A) | (I)LQ(W) | (Q)DK(T) |  | (E)RFF(S) | (Q)PTT(M) |
|  | (E)QN(Q) | (Y)IP(I) | (K)SC(Q) | (T)EI(P) |  | (K)YIP(I) | (T)TMA(R) |
|  | (Q)NQ(E) | (I)PI(Q) | (S)CQ(A) | (E)IP(T) |  | (Y)VLS(R) | (M)AIP(P) |
|  | (N)QE(Q) | (P)IQ(Y) | (R)HP(H) | (I)AS(G) |  | (S)RYP(S) | (S)TPT(T) |
|  | (Q)EQ(P) | (L)SR(Y) | (H)PH(P) | (V)AT(L) |  | (K)PVA(L) | (T)PTT(E) |
|  | (Q)PI(R) | (N)NQ(F) | (P)HP(H) | (T)LE(D) |  | (F)LPY(P) | (T)EAV(E) |
|  | (C)EK(D) | (F)LP(Y) | (H)PH(L) | (V)IE(S) |  | (K)PAA(V) | (E)AVE(S) |
|  | (E)KD(E) | (Y)YA(K) | (A)IP(P) | (P)EI(N) |  | (P)AAV(R) | (E)DSP(E) |
|  | (E)RF(F) | (A)VR(S) | (K)KN(Q) | (S)TA(V) |  | (R)SPA(Q) | (E)SPP(E) |
|  | (F)FS(D) | (V)RS(P) | (K)NQ(D) |  |  | (Q)VLS(N) |  |

**TABLE S2** Dipeptides and tripeptides identified from peak 5 from the casein hydrolysate

| **Location** | **Dipeptide** | **Dipeptide** | **Dipeptide** | **Tripeptide** | **Tripeptide** | **Tripeptide** |
| --- | --- | --- | --- | --- | --- | --- |
| ***α*-S1 casein** | (N)EL(S) | (I)HA(Q) | (D)IP(N) | (N)LLR(F) | (E)RYL(G) | (F)RQF(Y) |
|  | (E)EI(V) | (I)GV(N) | (N)PI(G) | (L)LRF(F) | (Q)LLR(L) | (Q)LDA(Y) |
|  | (Y)LE(Q) | (V)NQ(E) | (M)PL(W) | (L)RFF(V) | (L)LRL(K) | (V)PLG(T) |
|  | (Q)LE(I) | (Q)EL(A) | (P)LW | (F)VAP(F) | (K)EGI(H) | (D)APS(F) |
|  | (L)EI(V) | (P)EL(F) |  | (P)FPE(V) | (E)LFR(Q) | (N)PIG(S) |
|  | (H)SM(K) | (V)PL(G) |  | (K)EDV(P) | (L)FRQ(F) |  |
| ***α*-S2 casein** | (E)HV(S) | (I)NQ(F) | (P)WI(Q) | KNT(M) | (A)EVA(T) | (F)ALP(Q) |
|  | (E)KN(M) | (V)PI(T) | (V)IP(Y) | (E)KNM(A) | (N)AVP(I) | (V)IPY(V) |
|  | (V)VR(N) | (E)KN(R) | (Y)VR(Y) | (F)CKE(V) | (I)TPT(L) | (V)RYL |
|  | (V)AT(E) | (K)FA(L) |  | (R)NAN(E) | (N)SKK(T) |  |
|  | (N)EI(N) | (A)LP(Q) |  | (S)AEV(A) | (K)LTE(E) |  |
| ***β*-casein** | (R)EL(E) | (I)HN(S) | (L)PL(P) | (P)GEI(V) | (S)QSK(V) |  |
|  | (E)LE(E) | (S)LP(Q) | (P)LP(L) | (Q)SLV(Y) | (K)AVP(Y) |  |
|  | (E)EL(N) | (P)QN(I) | (L)PL(L) | (F)PGP(I) | (A)FLL(Y) |  |
|  | (G)EI(V) | (N)IP(P) | (F)PP(Q) | (P)GPI(H) | (V)LGP(V) |  |
|  | (I)NK(K) | (I)PP(L) | (M)PI(Q) | (A)MAP(K) |  |  |
|  | (K)IE(K) | (P)PL(T) | (Q)AF(L) | (V)EPF(T) |  |  |
|  | (D)EL(Q) | (V)PP(F) | (P)VR(G) | (T)DVE(N) |  |  |
|  | (P)FA(Q) | (M)GV(S) |  | (Q)SVL(S) |  |  |
|  | (G)PI(H) | (H)LP(L) |  | (S)VLS(L) |  |  |
| ***κ*-casein** | (E)QN(Q) | (A)VR(S) | (V)AT(L) | (R)CEK(D) | (F)LPY(P) | (S)TPT(T) |
|  | (Q)NQ(E) | (A)IP(P) | (T)LE(D) | (D)ERF(F) | (R)SPA(Q) | (T)PTT(E) |
|  | (Q)PI(R) | (I)PP(K) | (V)IE(S) | (E)RFF(S) | (Q)VLS(N) | (T)EAV(E) |
|  | (Y)IP(I) | (K)KN(Q) | (S)PP(E) | (K)YIP(I) | (T)VPA(K) | (E)AVE(S) |
|  | (I)PI(Q) | (K)NQ(D) | (P)EI(N) | (Y)VLS(R) | (Q)PTT(M) | (E)DSP(E) |
|  | (N)NQ(F) | (T)EI(P) | (S)TA(V) | (S)RYP(S) | (M)AIP(P) | (E)SPP(E) |
|  | (F)LP(Y) | (E)IP(T) |  | (K)PVA(L) | (K)TEI(P) |  |

**TABLE S3** Dipeptides and tripeptides identified from peak 6 from the casein hydrolysate

| **Location** | **Dipeptide** | **Dipeptide** | **Tripeptide** | **Tripeptide** |
| --- | --- | --- | --- | --- |
| ***α*-S1 casein** | (R)PK(H) | (L)LR(L) | (L)LRF(F) | (E)LFR(Q) |
|  | (L)PQ(E) | (L)RL(K) | (L)RFF(V) | (L)FRQ(F) |
|  | (L)LR(F) | (E)RL(H) | (F)PEV(F) | (F)RQF(Y) |
|  | (V)AP(F) | (D)AP(S) | (A)QQK(E) | (P)SGA(W) |
|  | (L)SK(D) |  | (M)IGV(N) | (S)DIP(N) |
| ***α*-S2 casein** | (K)NT(M) | (N)RL(N) | (P)IVL(N) |  |
|  | (E)HV(S) | (I)SQ(R) | (F)ALP(Q) |  |
|  | (I)SQ(E) | (L)PQ(Y) |  |  |
|  | (P)SK(E) | (M)KP(W) |  |  |
|  | (F)CK(E) | (I)QP(K) |  |  |
|  | (F)PQ(Y) | (Q)PK(T) |  |  |
|  | (N)SK(K) |  |  |  |
| ***β*-casein** | (T)RI(N) | (E)SQ(S) | (N)VPG(E) | (Q)EPV(L) |
|  | (F)QS(E) | (S)QS(L) | (E)QQQ(T) | (P)VLG(P) |
|  | (T)QS(L) | (H)QP(H) | (N)SLP(Q) | (L)GPV(R) |
|  | (L)PQ(N) | (P)PQ(S) | (Q)TPV(V) | (P)IIV |
|  | (L)QP(E) | (P)QS(V) | (P)VVV(P) |  |
|  | (V)SK(V) | (L)SQ(S) | (Q)PEV(M) |  |
|  | (M)AP(K) | (S)QS(K) | (Y)PVE(P) |  |
|  | (A)PK(H) | (Q)SK(V) | (P)VEP(F) |  |
|  | (F)PK(Y) |  | (P)PTV(M) |  |
| ***κ*-casein** | (E)QP(I) | (V)PA(K) | (E)RFF(S) | (S)PEV(I) |
|  | (P)IR(C) | (A)KS(C) | (S)RYP(S) | (E)SPP(E) |
|  | (Q)KP(V) | (S)CQ(A) | (Y)QQK(P) |  |
|  | (A)KP(A) | (A)QP(T) | (V)ALI(N) |  |
|  | (K)PA(A) | (I)NT(I) | (N)TVP(A) |  |
|  | (S)PA(Q) | (I)NT(V) | (M)AIP(P) |  |
|  | (S)NT(V) |  | (I)ASG(E) |  |


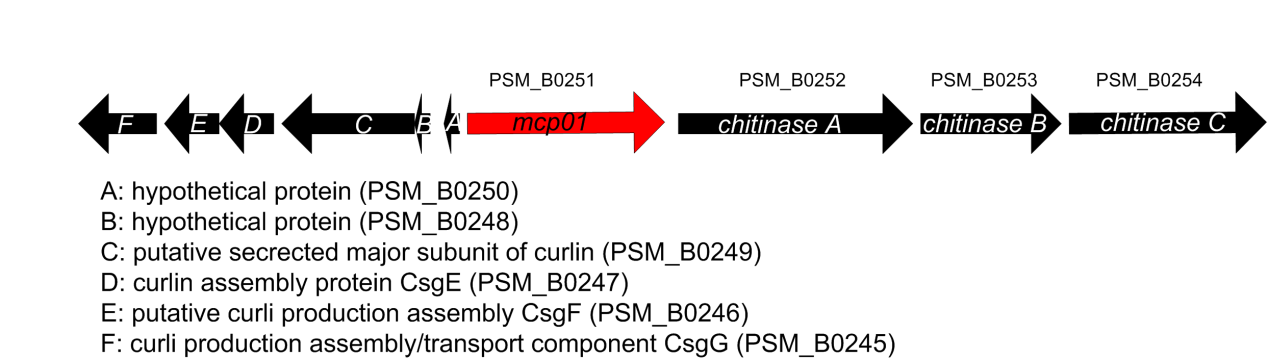


**FIGURE S1** Gene organization in the surrounding of gene *mcp01* in SM9913.
